# Quantitative Analysis of Adulterations in Oat Flour by FT-NIR Spectroscopy, Incomplete Unbalanced Randomized Block Design, and Partial Least Squares

**DOI:** 10.1155/2014/393596

**Published:** 2014-07-20

**Authors:** Ning Wang, Xingxiang Zhang, Zhuo Yu, Guodong Li, Bin Zhou

**Affiliations:** School of Material Science and Engineering, Tianjin Municipal Key Lab of Fiber Modification and Functional Fiber, Tianjin Polytechnic University, Tianjin 300389, China

## Abstract

This paper developed a rapid and nondestructive method for quantitative analysis of a cheaper adulterant (wheat flour) in oat flour by NIR spectroscopy and chemometrics. Reflectance FT-NIR spectra in the range of 4000 to 12000 cm^−1^ of 300 oat flour objects adulterated with wheat flour were measured. The doping levels of wheat flour ranged from 5% to 50% (w/w). To ensure the generalization performance of the method, both the oat and the wheat flour samples were collected from different producing areas and an incomplete unbalanced randomized block (IURB) design was performed to include the significant variations that may be encountered in future samples. Partial least squares regression (PLSR) was used to develop calibration models for predicting the levels of wheat flour. Different preprocessing methods including smoothing, taking second-order derivative (D2), and standard normal variate (SNV) transformation were investigated to improve the model accuracy of PLS. The root mean squared error of Monte Carlo cross-validation (RMSEMCCV) and root mean squared error of prediction (RMSEP) were 1.921 and 1.975 (%, w/w) by D2-PLS, respectively. The results indicate that NIR and chemometrics can provide a rapid method for quantitative analysis of wheat flour in oat flour.

## 1. Introduction

Food adulteration and fraud have been a common problem in food production since ancient times. Food adulteration is economically motivated and is performed by the addition, substitution, or removal of food ingredients, for example, replacing or diluting high-cost ingredients with cheaper ones [1, 2]. It is an issue that concerns not only consumers, but also food producers, sellers, regulatory agencies, and even the entire food industry chain. For consumers, food adulterations have caused growing concern about health risks, as well as the food quality and nutrition value. A notorious and common phenomenon is the adulterations of raw food materials, which can not only influence the quality of raw materials but also cause potential crisis in further processed foods [3, 4].

Oat is widely utilized for human consumption and food industrial uses. Due to its high nutritional value and characteristic flavor, oat flour plays an important role in the breakfast cereals group and other processed foods as an alternative or supplement to the ordinary wheat flour [5]. As a nonstaple cereal, the yield of oat is much less than wheat, so oat flour is more expensive than wheat flour. For producers and sellers, it is economically profitable to add wheat flour to oat flour. Because the appearances and physical and chemical properties of wheat and oat flours are very similar, rapid and effective methods are required to analyze the adulterations.

For food analysis and quality control, NIR spectroscopy has demonstrated some advantages, including less sample treatment, reduced analysis time and cost, and the feasibility for nondestructive analysis and online analysis. NIR spectroscopy has been widely used for analysis of grains and cereals. Hurburgh et al. [6] combined NIR spectroscopy and principal component analysis to discriminate transgenic grains and nontransgenic grains. Munck et al. [7] applied the NIRS technology to distinguish barley flour with different levels of lysine amino acids. NIR was also successfully used to distinguish corn samples of different genotypes [8, 9]. For quantitative analysis, NIR technology has provided a rapid tool for analysis of different constituents or quality parameters in grain products, including corn dry-milling quality [10], protein content in wheat kernels [11], the ratio of starch amylose content to total grain in corn [12], undried rough rice constituent content [13], kernel rots and mycotoxins in maize [14], and protein, moisture, dry mass, hardness, and other residues of wheat [15] and so on [16].

Considering the large number of samples in market shelf and small private retailers, NIR is a convenient and economic technique for analysis of adulterations in oat flour. This paper was aimed at developing a rapid method for analysis of potential wheat flour added to oat flour using NIR spectrometry and chemometrics. Considering the composition variations of oats from different producing areas, an incomplete unbalanced randomized block design [17] was performed to ensure the generalization performance of multivariate calibration models.

## 2. Materials and Methods

### 2.1. Collection and Preparation of Samples

Pure oat flour and wheat flour objects were made from intact grains. A set of oat and wheat kernels harvested in 2013 were collected from domestic markets. Oat kernels were produced in Hebei (15), Henan (17), Gansu (17), Shanxi (18), and Qinghai (13). Wheat kernels were produced in Henan (11), Hebei (10), Jiangsu (8), Anhui (10), Shandong (11), Shanxi (7), and Heilongjiang (8). The kernels were dried in the sun and milled by a crusher. All the particles were filtered through a 200-mesh sieve.

Adulterated oat flour samples were made by mixing the oat flour with different levels of the wheat flour. The doping levels were 5%, 10%, 15%, 20%, 25%, 30%, 35%, 40%, 45%, and 50% (w/w). In order to obtain a representative nut not too large sample set, an incomplete and unbalanced randomized design [17] was performed. Because this paper was focused on quantitative analysis of wheat flour in oat flour, the producing areas of oat and wheat were considered to be two blocking factors. In this way, 120 adulterated objects were prepared for developing calibration models with 10 doping levels each having 12 objects. Another 100 adulterated objects (10 doping levels each having 10 objects) were prepared for model validation by mixing oat and wheat flour objects that were different from those used for preparation of training samples.

The NIR diffuse reflectance spectra of impacted powders were measured on a Bruker-TENSOR37 FTIR spectrometer (Bruker Optics, Ettlingen, Germany). The working range of spectrometer was 4000–12000 cm^−1^. The spectra were measured using a PbS detector with an internal gold background as the reference. The instrument resolution was 4 cm^−1^ and the scanning interval was 1.929 cm^−1^, so each spectrum contained 4148 wavelengths. Each spectrum was the average of 64 scans. For each object, three spectra were measured by stirring the powder and the average spectrum was taken.

### 2.2. Data Preprocessing and Multivariate Calibration

All the data preprocessing and chemometrics models were performed using MATLAB 7.0.1 (Mathworks, Sherborn, MA). Smoothing was used to remove random noise in the data and improve the signal-to-noise ratio (SNR). In this work the S-G polynomial fitting algorithm [18] was used for smoothing for its simplicity and effectiveness. Taking second-order derivative (D2) of spectra was performed to enhance spectral resolution and remove linear baseline shifts. The D2 spectra were also computed by S-G polynomial fitting algorithm because this method can avoid degradation of SNR compared with direct differencing. Standard normal variate (SNV) [19] transformation was performed to reduce the spectral variations caused by scattering and uneven sizes of particle.

Partial least squares (PLS) models were developed using the raw and preprocessed spectra. An important problem when performing PLS is the overfitting of models. In this paper, Monte Carlo cross-validation (MCCV) [20] was used to select the number of PLS components. MCCV can avoid the risk of overfitting by multiple random splitting of the training objects and having a higher percent of leave-out objects for prediction.

## 3. Results and Discussion

**Figure 1:**
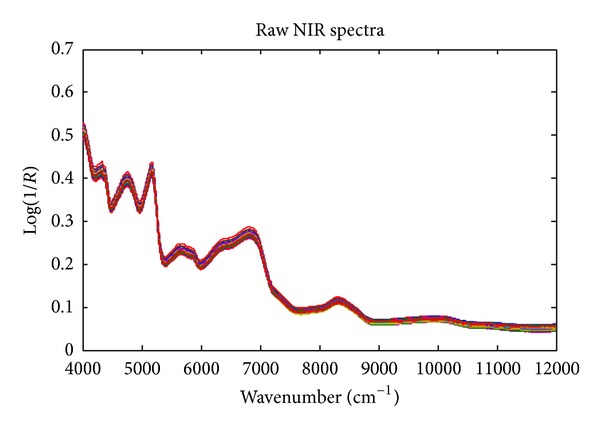
The raw NIR spectra of 120 adulterated oat flour objects with doping levels ranging from 5% to 50% (w/w).

The raw NIR spectra of 120 adulterated oat flour objects are shown in Figure 1. Some of the absorbance peaks can be assigned as follows [21]: (1) the peak at 4318 cm^−1^ caused by the combination absorbance of –CH_2_ deformation and various C–H stretching; (2) the wide peak at 4748 cm^−1^, overlapping of combination of C=O stretching and peptide group deformation and combination of N–H stretching and peptide group deformation; (3) the peak at 5167 cm^−1^, combination of O–H stretching and O–H deformation; (4) 5629 cm^−1^, the first overtone of symmetrical and asymmetrical C–H stretching in –CH_2_; (5) the wide peak at 6802 cm^−1^, overlapping of the first overtone of O–H stretching (~6900 cm^−1^) and the first overtone of N–H stretching (~6500 cm^−1^); (6) 8329 cm^−1^, the second overtones of C–H stretching in various groups; and (7) 9970 cm^−1^, the second overtones of N–H stretching or the third overtones of C–H stretching. The spectral interval 9000–12,000 cm^−1^ has no significant peaks, so this interval was not used for developing calibration models.

**Figure 2:**
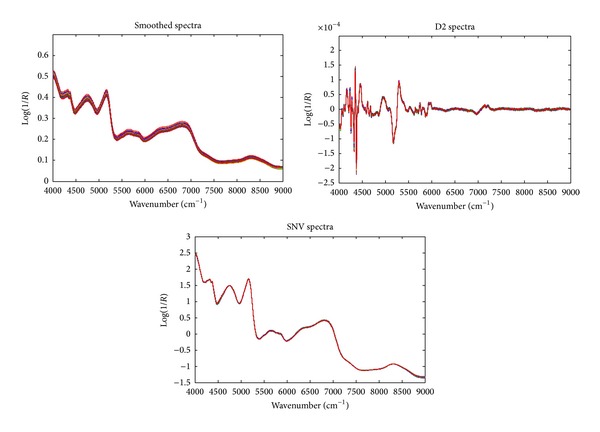
The smoothed, second-order derivative (D2) and standard normal variate (SNV) NIR spectra of 120 adulterated oat flour objects with doping levels ranging from 5% to 50% (w/w).

Smoothed, D2, and SNV spectra were shown in Figure 2. Seen from Figure 2, the D2 spectra can remove most of the backgrounds and the peak resolution was largely improved by taking D2 spectra. D2 spectra also obtained much detailed and high-frequency information. SNV transformation can remove most unwanted variations. Multivariate calibration models were developed with PLS to predict the levels of wheat flour. The number of PLS components was estimated using* F*-test of MCCV. In this work, random splitting of the training set was performed for 100 times and each time 70% of the training objects were used for developing a PLS model and 30% for prediction. The pooled predicted residual sum of squares (PRESS) was computed using different numbers of PLS components. Finally,* F*-test was performed to select the fewest PLS components with a PRESS value not significantly higher than the minimum PRESS value. As recommended by the original literature, the significance level of the* F*-test was set to be 0.25 [22, 23].

**Figure 3:**
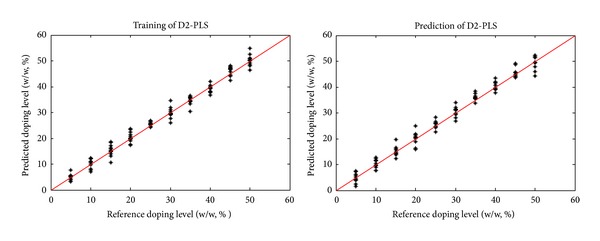
The training and prediction of contents of wheat flour in oat flour by three-component D2-PLS.

Based on differently preprocessed spectra (9000–4000 cm^−1^) the calibration and prediction results of PLS were demonstrated in Table 1. Seen from Table 1, the model complexity of D2-PLS and SNV-PLS was reduced by one component compared with the PLS model using raw data. Moreover, seen from the model complexity of PLS models (3 or 4 components), the mixtures of wheat flour and oat flour investigated in this work were not a simple two-component system and spectral variations caused by different geographical origins had made it necessary to perform multivariate calibration. The root mean squared error of MCCV (RMSEMCCV) value was slightly reduced by smoothing (2.274), taking D2 (1.921), and SNV transformation (1.981) compared with PLS with raw data (2.388). For prediction, the lowest root mean squared error of prediction (RMSEP) of 1.975 was obtained by D2-PLS model. The training and prediction results by D2-PLS are demonstrated in Figure 3. For all of the PLS models, the differences between RMSEP and RMSEMCCV values were insignificant, indicating that both the training and test sets obtained by IURB design were representative to include composition variations caused by different origins of oat and wheat flour.

## 4. Conclusions

Multivariate calibration models were developed by PLS for analysis of wheat flour in oat flour from different geographical origins. The results demonstrated that a three- or four-component PLS model can accurately predict the levels of wheat flour in oat flour. Moreover, IURB design was shown to be useful to obtain representative training and test sets to include the composition variations caused by different producing areas. The developed PLS models will have a good generalization performance and are useful for quantitative analysis of oat flour in domestic market.

## Figures and Tables

**Table 1:** Quantitative analysis of wheat flour in oat flour by PLS.

Preprocessing	*N* ^a^	RMSEMCCV (%)	RMSEC (%)	RMSEP (%)
Raw data	4	2.388	2.115	2.213
Smoothing	4	2.274	2.102	2.244
D2	3	1.921	1.781	1.975
SNV	3	1.981	1.842	2.054

^a^The number of significant PLS components.
